# High Throughput Micro-Well Generation of Hepatocyte Micro-Aggregates for Tissue Engineering

**DOI:** 10.1371/journal.pone.0105171

**Published:** 2014-08-18

**Authors:** Elien Gevaert, Laurent Dollé, Thomas Billiet, Peter Dubruel, Leo van Grunsven, Aart van Apeldoorn, Ria Cornelissen

**Affiliations:** 1 Tissue Engineering Group, Ghent University, Ghent, Belgium; 2 Liver cell biology laboratory, Vrije Universiteit Brussels (VUB), Brussels, Belgium; 3 Polymer Chemistry and Biomaterials Research Group, Ghent University, Ghent, Belgium; 4 Department of Developmental Bioengineering, University of Twente, Enschede, the Netherlands; Osaka University, Japan

## Abstract

The main challenge in hepatic tissue engineering is the fast dedifferentiation of primary hepatocytes in vitro. One successful approach to maintain hepatocyte phenotype on the longer term is the cultivation of cells as aggregates. This paper demonstrates the use of an agarose micro-well chip for the high throughput generation of hepatocyte aggregates, uniform in size. In our study we observed that aggregation of hepatocytes had a beneficial effect on the expression of certain hepatocyte specific markers. Moreover we observed that the beneficial effect was dependent on the aggregate dimensions, indicating that aggregate parameters should be carefully considered. In a second part of the study, the selected aggregates were immobilized by encapsulation in methacrylamide-modified gelatin. Phenotype evaluations revealed that a stable hepatocyte phenotype could be maintained during 21 days when encapsulated in the hydrogel. In conclusion we have demonstrated the beneficial use of micro-well chips for hepatocyte aggregation and the size-dependent effects on hepatocyte phenotype. We also pointed out that methacrylamide-modified gelatin is suitable for the encapsulation of these aggregates.

## Introduction

Upon isolation of their native micro-environment, hepatocytes rapidly lose their viability and metabolic functions. [1] This limits or prevents their use for clinical, engineering and research purposes. The prolonged maintenance of hepatocyte phenotype could e.g. lead to the development of an engineered donor tissue, bioartificial liver device, more efficient transplantation methods, the development of more reliable *in vitro* models thereby improving drug toxicity screening, liver disease research and many more. [2], [3]

To improve hepatocyte performance many strategies have been addressed. One approach is the cultivation of hepatocytes as aggregates, allowing enhanced cell-cell contacts in a three dimensional context. Cell-aggregates can be considered as micro-tissues and are more representative for liver tissue than conventional two dimensional cell cultures. Different techniques such as static cell culture on non-adherent surfaces or micro patterned surfaces, hanging drop and rotary culture systems have been used for creating hepatocyte aggregates with variable dimensions. [4] These studies have demonstrated that the cultivation of hepatocytes as aggregates improves many of their metabolic functions such as cytochrome P450 activity, albumin secretion, urea production, glutathione S-transferase activity, etc. [5], [6] [4], [7]

Most living tissues, including the liver, are composed of repeating cellular units on a scale of hundreds of microns. Artificially generated three dimensional cell aggregates comprising hepatocytes could potentially serve as functional building blocks for the creation of larger constructs. [8] Assembly of these building blocks into larger constructs with more relevant dimensions, can be obtained by self-assembly or assembly in a more directed way using biomaterials to guide this process. [9], [10] In this context, the immobilization and organization of the aggregates at high density, while allowing mass transport of nutrients and metabolites for hepatocyte survival and function, could be useful. [4],[8]

In this study, we used a microwell-based tissue culture platform to generate large quantities of hepatocyte aggregates of predefined dimensions and shapes. We observed that aggregate dimensions affect the overall performance of the hepatocytes and selected the most optimal aggregation parameters. Combining these well defined primary hepatocyte aggregates with a gelatin methacrylamide hydrogel allows the encapsulated aggregates to maintain a proper hepatocyte phenotype. The present work demonstrates the generation of large amounts of relatively small aggregates that can be organized into larger constructs by encapsulation in a gelatin hydrogel. [11], [12]

## Materials and Methods

The study protocol was approved by the Ethical committee for Animal Care and Use of Ghent University (permit number ECD 12/42). Institutes of Health principles of laboratory animal care (NIH) were followed.

### 2.1. Cell culture and isolation of primary hepatocytes

HepG2 cells were maintained in DMEM Glutamax supplemented with 10% v/v FBS, 50 U/ml penicillin and 50 µg/ml streptomycin, all provided by Life Technologies.

Primary mouse hepatocytes were isolated from adult mouse livers (ICR CD-1 mice, 8–14 weeks of age, Harlan Laboratories). The animals were anaesthetized with ketamin/xylazin and died during the perfusion procedure. The hepatocytes were isolated from the liver using a two-step collagenase perfusion method, followed by percoll gradient purification as described by Conçalves *et al* [13]. After isolation, cells were immediately seeded in micro-wells, or well plates coated with 0.1% collagen (BD Bioscienses), in order to compare three dimensional with two-dimensional cell culture.

Primary hepatocytes were cultured in William's E medium (Life Technologies), supplemented with L-glutamine (292 mg/ml) (Life Technologies), glucagon (7 ng/ml) (sigma), insulin (0.5 µg/ml) (sigma), hydrocortisone (25 µg/ml), EGF (10 ng/ml), 10% v/v FBS (Life Technologies), 50 U/ml penicillin (Life Technologies) and 50 µg/ml streptomycin (Life Technologies).

Both the HepG2 cells and the primary hepatocytes were maintained in a humidified 5% CO_2_-containing atmosphere at 37°C.

### 2.2. Micro-well synthesis and micro-aggregate formation

To produce the non-adherent agarose micro-wells, sterilized powder Ultrapure Agarose (Life Technologies) was dissolved (3% w/v) and heated in PBS. The liquid agarose solution was added to a tailor-made, negative polydimethylsiloxane mold (PDMS, as described previously [14], [15]) and left to solidify at RT. After cooling, the gels were separated from the moulds and subsequently transferred into 12 well culture plates. Single cell suspensions of various densities (detailed information in table 1 and table 2) were seeded in the chip containing the micro-wells. After seeding, formation of micro-aggregates was supported by centrifugation for 1 min at 1500 rpm to let the cells settle into the bottom of the micro-wells. Culture medium was replenished 24 h after seeding and cells were left in culture for 3 days to allow formation of stable micro-aggregates. The aggregates were harvested at different time points, as indicated in the results section.

**Table 1:** Overview of the applied parameters for HepG2 cells and primary hepatocytes and their characteristics.

Seeded number of cells/chip	Mean aggregate diameter (µm)	Mean number of cells/aggregate	Mean aggregate volume (µm^3^)	Diameter of micro-wells in the chip
1000000	307±13	629	15120449	400 µm
750 000	297±9	472	13745009	400 µm
500 000	261±15	314	9255967	400 µm
250 000	231±7	157	6479261	400 µm
1 000 000	166±11	87	2408105	200 µm
750 000	157±9	175	2010823	200 µm
500 000	142±7	262	1489732	200 µm
250 000	116±9	349	810959	200 µm

**Table 2:** Overview of the applied parameters for primary hepatocytes and their characteristics.

Seeded number of cells/chip	Mean aggregate diameter (µm)	Mean number of cells/aggregate	Mean aggregate volume (µm^3^)	Diameter of micro-wells in the chip
150 000	185±16	94	3336782	400 µm
75 000	90±13	47	385917	400 µm
150 000	95±13	52	452331	200 µm
75 000	60±8	26	111241	200 µm

Aggregate diameter, perimeter (p) and aggregate area (A) was determined using Xcellence image software. Subsequently circularity was calculated using the formula *f_circ_* = (4πA)/p^2^.

Aggregate volume was determined based on the diameter and the volume formula of a spherical object. The number of cells per aggregates was estimated, based on the number of microwells per chip and the amount of seeded cells per chips. All aggregate characteristics and parameters are represented in table 1 and table 2.

### 2.3. Aggregate encapsulation

Gelatin methacrylamide with a degree of substitution (DS) of 65% was used to encapsulate aggregates as described earlier.[16] Briefly, aggregates (2300 aggregates/ml) were mixed in dissolved gelatin (10% w/v in PBS) with 2 mol % Irgacure 2959 photo-initiator, as calculated relative to the (photo-sensitive) methacrylamide side groups. The aggregate-gelatin mixture was transferred (200 µl/well) into 48 well plates (Greiner Bio-one) and left for physical gelation during 30 min. Photocross-linking was carried out by exposure to UV-A light (365 nm, 2 mW/cm^2^, UVP Inc.) for 10 min. After cross-linking, culture medium was added and refreshed every 24 h.

### 2.4. Cell viability

To assess the cell viability, aggregates were washed twice with PBS and incubated for 10 minutes with 2 µg/ml calcein-AM (AnaSpec) and 2 µg/ml propidium iodide (PI) (Sigma). After washing in PBS for 10 min, cell viability was evaluated by determining the ratio of green (live) versus red (dead) cells using an inverted fluorescence microscope (Olympus IX81) equipped with Xcellence software (Olympus).

### 2.5. Histology

Samples were fixed (4% paraformaldehyde) and embedded in paraffin. Five µm thick sections were stained with hematoxilin and eosin (HE), Periodic acid Schiff (PAS) or immunostained (IHC), all at room temperature.

For IHC staining, endogenous peroxidase was quenched using 3% v/v H_2_0_2_ for 1 h, and a 30 min treatment with blocking reagent (1% w/v BSA, 0.2% v/v Tween 20 in PBS) was performed. The samples were incubated with the primary antibody (dilution 1∶100) for 2 h and subsequently with the secondary antibody (dilution 1∶200) for 30 min. A goat anti-mouse albumin antibody (p20, Santa Cruz) and a rabbit anti-mouse HNF4α (H-171, Santa Cruz) was used as primary antibody for albumin and HNF4α respectively. A 3,3-diaminobenzidine tetrahydrochloride substrate was used to visualize the horse radish peroxidase coupled secondary antibody. After hematoxylin staining and mounting, the samples were visualized under the microscope. For HNF4α the whole staining procedure was preceded by antigen retrieval using citrate buffer (pH 6.00).

For PAS stain, the samples were treated with 1% v/v periodic acid for 15 min. After washing with PBS, samples were exposed to Schiff reagent (Sigma) for 30 minutes in the dark. After rinsing in water, the slides were stained with hematoxylin and mounted.

For transmission electron microscopy, aggregates were fixed with glutaraldehyde, postfixed with OsO_4_ and embedded in epoxy resin. Sections (70 µm thick) were evaluated by a Jeol 1200 EX II electron microscope.

### 2.6. Cytochrome activity

Cyp3A4 activity of the primary hepatocyte aggregates was analyzed using a P450-Glo-CYP3A assay (Promega) according to the manufacturer's protocol for cell-based assays. After 72 h culture in the micro-wells, aggregates were exposed for 1 h to culture media containing luciferin-IPA (1∶1000). After 1 h, an equal volume of the liquid was transferred to a 96 well plate and incubated with an equal volume of detection reagent. After 20 min, luciferase activity was detected using a Wallac 1420 Victor^3^ multilabel counter (Perkin Elmer). The detected luminescence was normalized for the amount of cells (MTT assay). Background subtraction was performed with culture medium considered as negative control.

### 2.7. Albumin secretion

To assess albumin secretion, media samples were collected at different time points. After omitting cross-reactivity for bovine albumin, the amount of secreted albumin was determined using a mouse albumin ELISA quantitation kit or a human albumin ELISA quantitation kit (Bethyl laboratories, Inc., UK) and normalized for the amount of cells using an MTT assay.

### 2.8. MTT assay

The hepatocytes were incubated with a 0.5 mg/ml solution of MTT (Calbiochem) in culture medium for four hours in the dark in a 5% CO_2_ incubator at 37°C. After discarding the MTT solution, the formed formazan crystals were dissolved in isopropanol-0.04N HCl supplemented with 1% v/v Triton X100 (Sigma). Subsequently the absorbance was measured at 580 nm using an EL800 Universal microplate reader (BioTek instruments Inc.) and compared to a standard curve.

### 2.9. Real Time Polymerase chain reaction (RT-PCR)

Total RNA was extracted from hepatocyte aggregates using TRI Reagent (Sigma-aldrich) and treated with DNAse digestion kit (Invitrogen). RT Core Kit (Eurogentec) was used to synthesize cDNA, according to the manufacturer's protocol. A 7500 Fast Real-Time PCR system (Applied Biosystems) and a SYBR Green PCR kit (Eurogentec) were used according to the manufacturer's instructions and protocols. GAPDH expression was used as stable housekeeping marker for reference. The relative gene fold changes were determined by the 2^−ΔΔCt^ method. For comparative gene expression analysis of free floating aggregates gene expression was compared to expression levels of freshly isolated hepatocytes. Additionally, to assess the effect of hydrogel embedding, encapsulated hepatocyte aggregates were compared to aggregates before encapsulation (day 3). Primer sequences are depicted in table S1 and S2.

### 2.10. Statistical Analysis

Differences between groups were explored by one-way ANOVA, followed by a Student *t*-test using the statistical package GraphPad Prism 4 (San Diego California, USA).

## Results

### 3.1. Development of micro-aggregates with controlled size

Tailor-made PDMS molds were used to generate agarose microwell-containing chips. Briefly, liquid agarose was poured on top of PDMS molds containing micro-sized cylindrical sticks. After solidifying, the PDMS molds were removed from the agarose resulting in agarose chips containing micro-sized wells. (illustrated fig. S1) The fabrication of these micro-well-containing chips resulted in micro-well array chips containing 2865 or 1585 wells with a diameter of 0.2 or 0.4 mm respectively in agarose. After fabrication, the chips were placed in a 12 well plate and used for controlled hepatocyte aggregation.

First, the micro-aggregation procedure was optimized using HepG2 cells. Although this human hepatocellular carcinoma cell line is less representative of the *in vivo* situation, these cells are user-friendly, robust and aggregate easily while maintaining some hepatocyte specific functions. Subsequently the procedure was adapted and performed using primary mouse hepatocytes. The micro-aggregation behavior of and its effect on these cells was evaluated and compared.

**Figure 1:**
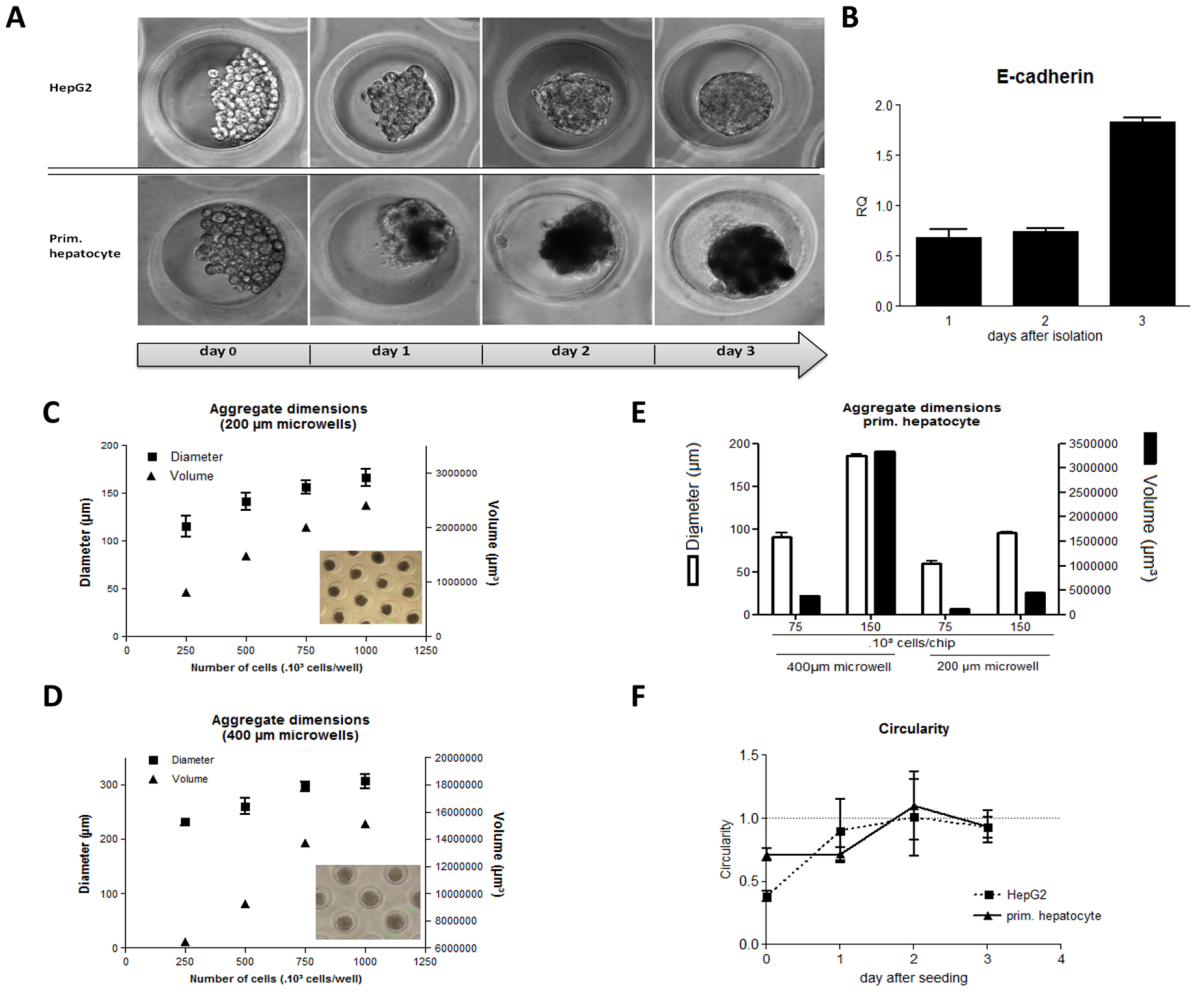
Formation of hepatocyte micro-aggregates in agarose microwells. (**A**) Aggregate formation over time for both HepG2 cells and primary hepatocytes. (**B**) E-cadherin gene expression in primary hepatocytes during aggregate formation (**C–D**) Correlation between number of cells, aggregate diameter (squares) and volume (triangles) for the formation of HepG2 cell aggregates in 200 (C) and 400 µm (D) microwells, n = 3. (**E**) Correlation between number of primary cells, aggregate diameter (solid line) and volume (dotted line) for the formation of primary hepatocyte aggregates in 200 (white squares) and 400 µm (black squares) microwells, n = 3. (**F**) circularity of aggregates during culture for HepG2 cells (squares) and primary hepatocytes (triangles), n = 3.

For both cell types, aggregate formation started from day 1 (fig. 1A). In addition, we observed that stable aggregates were formed within 3 days, which was accompanied by an increase in E-cadherin expression (fig. 1B). By varying parameters such as micro-well diameter, cell number and cell type, aggregates with different dimensions were obtained (fig. 1C–E). All applied parameters in relation to and affecting aggregate dimension are listed in tables 1 and 2. Aggregate diameters (φ) varied between 100 and 300 µm for HepG2 cells and between 50 to 200 µm for primary hepatocytes. The linear relation between aggregate volume and cell number observed in fig.1C–D, the constant circularity values starting from day 2 (fig. 1F) and microscopic observation indicate that the aggregates were spherical and uniform in size.

### 3.2. Hepatocyte specific performance is enhanced and is size dependent in micro-aggregates

For HepG2 aggregates, size dependent correlation of hepatocyte gene expression markers such as albumin, HNF4a and TTR was observed (fig. S6) and albumin secretion (fig. S7) was clearly enhanced for all conditions. It appeared that HepG2 cells are not very sensitive for variations in aggregate size shortly after aggregate formation (day 3, fig. S2), however 7 days after aggregate formation the smaller aggregates (φ≤231±7 µm) maintained higher hepatocyte-specific gene expression levels than the larger aggregates (φ≥261±15 µm). At this time point a substantial amount of dead cells was observed in the center of the larger (φ≥261±15 µm diameter) aggregates (fig. S3).

**Figure 2:**
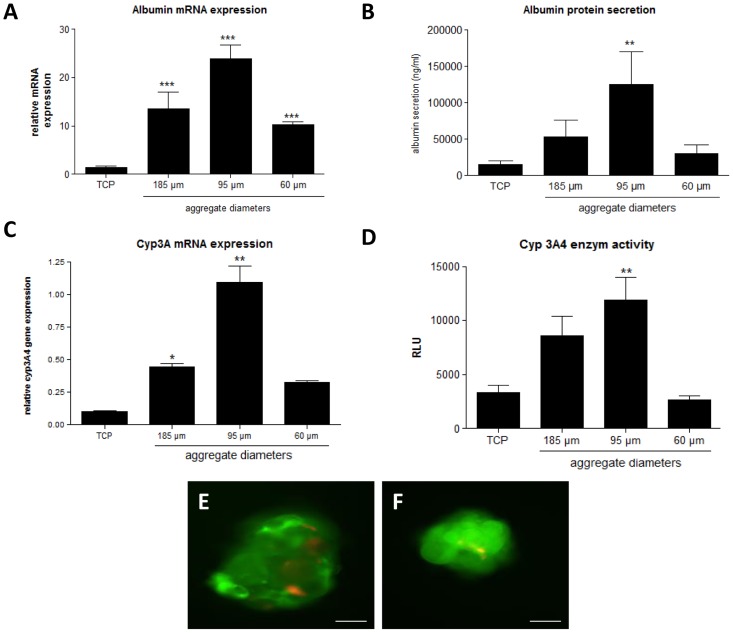
Evaluation of hepatocyte phenotype for different micro-aggregate sizes. (**A**) Gene expression of albumin. Gene expression levels of albumin were determined for different micro-aggregation conditions and compared to cells cultured on tissue culture plastic (TCP). (**B**) Albumin secretion at day 3 after isolation for different micro-aggregation conditions. Data are mean ± SD, n = 3, **p<0,01; compared to cells cultivated as monolayers (TCP). (**C**) Gene expression of CypA3. Gene expression levels of Cyp3A for different aggregate dimensions compared to cells cultured on tissue culture plates (TCP). Gene expression of Cyp3A was determined after 72 hours of cultivation in the micro-wells. Data are mean RQ ± SD, n = 3. *p<0.05; **p<0.01 (**D**) Induced cytochrome 3A4 activity in aggregates of different diameter versus cells cultured on tissue culture plastic. Cyp3A4 activity was quantified after treatment with an inducing agent (hydrocortisone) using a luciferase based assay after 3 days of isolation. Data are mean ± SEM, n = 3. *p<0,05; **p<0,01; ***p<0,001 compared to cells cultured as monolayers (TCP). (**E–F**) Live/dead stain of micro-aggregates with diameters of 200 µm (E) and 100 µm (F). Dead cells are stained red, while viable cells are stained green. *scale bar = 50* *µm*.

The gene expression and protein secretion of albumin, an important serum protein produced by the liver, was evaluated and normalized using primary hepatocyte aggregates and compared to primary hepatocytes plated on collagen 1 coated tissue culture plates (TCP) 3 days after isolation (fig. 2 A–B). In some aggregates both gene expression as well as protein secretion was significantly (*p*<0.05) enhanced compared to the control culture. However, clear differences in both secretion (fig. 2B) and gene expression (fig. 2A) were observed between aggregates of different sizes with a significant enhancement of both secretion (*p*<0.01) and gene expression (*p*<0.001) for 95±13 µm diameter aggregates. Analysis of the albumin secretion at day 7 after isolation indicated a general drop in albumin secretion for all conditions. However the differences between aggregates of different sizes remained and the 95±13 µm aggregates maintained the highest albumin secretion levels. (fig. S4)

A significant (*p*<0.05) improvement, compared to cells cultured as monolayers, in both cyp3A enzyme activity (fig. 2D) and gene expression (fig. 2C) was noticed for the 95.2±12.5 µm and 185±16 µm aggregates, while this improvement is less clear for the 60±8 µm aggregate, 3 days after isolation. Different aggregate sizes lead to different cyp3A performance with the medium sized aggregates (φ 95±13 µm, obtained by seeding 150 000 cells in the 200 µm micro-well) displaying the highest activity and the smallest aggregates (φ 60±8 µm) showing the lowest activity 3 days after isolation. The cyp3A4 enzyme activity at day 7 (fig. S5) showed a decrease for all conditions and for cells cultivated as monolayers the activity was not detectable. For the aggregates, the 95±13 µm aggregates displayed the highest cyp3A4 activity when compared to the 185±16 µm and 60±8 µm aggregates.

It was clear that hepatocyte phenotype depends on aggregate parameters and increasing aggregate dimensions did not necessarily lead to better performance. Live/dead staining of the different aggregates indicated excellent cell viability in the aggregates, however more dead cells were observed in the largest aggregates (φ≥185±16 µm, fig. 2E) when compared to the other aggregates (fig. 2F). This observation could indicate nutritional/waste diffusion limitations and explain why no clear relation between hepatocyte performance and aggregate size was observed.

### 3.3. Long term hepatocyte function is affected by aggregate dimensions

**Figure 3:**
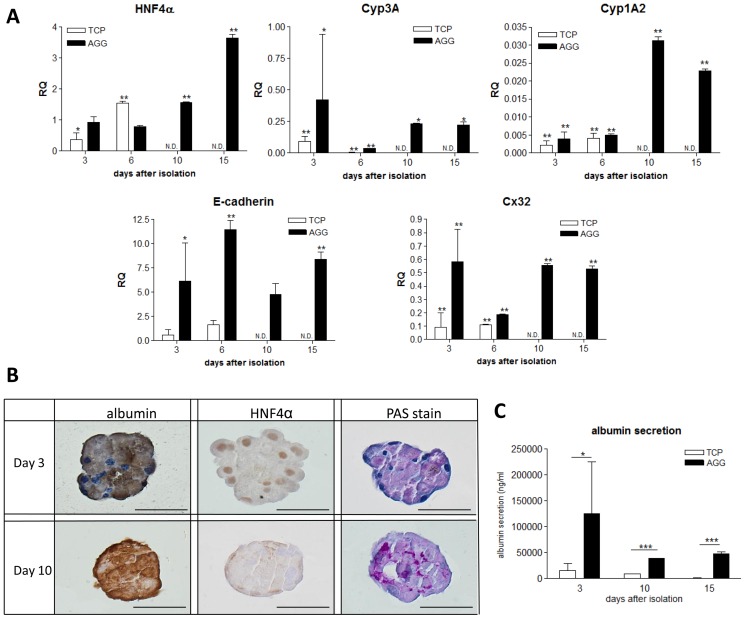
Evaluation of hepatocyte phenotype maintenance in micro-aggregates. (**A**) Gene expression analysis using real-time PCR. Gene expression of HNF4α, Cyp3A, Cyp1A2, E-cadherin and connexin 32 (Cx32) were determined for cells cultured on tissue culture plastic (white bars, TCP) and aggregates (black bars, AGG) at day 3, 6, 10 and 15 after isolation. The gene expression was normalized using GAPDH as stable housekeeping gene and related to freshly isolated hepatocytes (value = 1). Data are mean RQ ± SD, compared to freshly isolated, non cultured hepatocytes, n = 3. *p<0.05; **p<0.01. (**B**) Albumin, HNF4α, and PAS staining to visualize glycogen storage day 3 and day 10. *scale bar = 50* *µm*. (**C**) Albumin secretion of plated cells and microaggregates determined by ELISA. Albumin secretion during 24 hours at day 3, 10 and 15 was evaluated for cells cultured on tissue culture plastic (white bars, TCP) and aggregates (black bars, AGG). * p<0.05, ***p<0.001

Since aforementioned results indicated that 95±13 µm diameter primary hepatocyte aggregates performed most optimal, these were further evaluated. The hepatocyte specific function (fig. 3A) of two-dimensional cultured hepatocytes and hepatocyte aggregates were compared to freshly isolated non-cultured hepatocytes over time. At day 10 and 15, no expression of the genes of interest was detected for two-dimensional cultured cells (fig. 3A, TCP N.D.). For all examined genes, aggregates displayed a clear and significant upregulation of albumin, connexin32 (Cx32), hepatocyte nuclear factor 4α (HNF4α), E-cadherin, cyp3A, cytochrome 1A2 (cyp1A2) compared to cells in monolayers. The gene expression of HNF4α, an important transcription factor for the expression of hepatocyte specific genes, was maintained at a similar level in the aggregate as in freshly isolated hepatocytes, and increased over time. Gene expression of Cx32, indicative of gap junctions between cells, and cyp3A, indicative of active phase I metabolism, had decreased. Gene expression of E-cadherin, important for cell-cell adhesion and required for aggregate formation and stability, was upregulated in aggregates when compared to freshly isolated cells and remained stable during 15 days of culture.

The synthesis of albumin and HNF4α protein in the 100 µm aggregate was visualized using IHC staining and storage of glycogen was determined by PAS staining. Albumin secretion was significantly enhanced (2-fold increase) as compared to the plated cells at all time points.

Transmission electron microscopy (fig. S8) showed the presence of narrow contacts (desmosomes and gap junctions) between the cells and normal cell morphology with numerous mitochondria, abundant RER within the cytoplasm of hepatocytes cultured for 10 days in aggregates.

### 3.4. Micro-aggregates remain viable and functional upon encapsulation in gelatin

For tissue engineering purposes, the creation of larger constructs might be desirable (i.e. for the creation of bio-artificial liver devices, incorporation in a bioreactor). To organize these micro-aggregates at a larger scale, encapsulation and immobilization in a hydrogel could be a useful approach.

**Figure 4:**
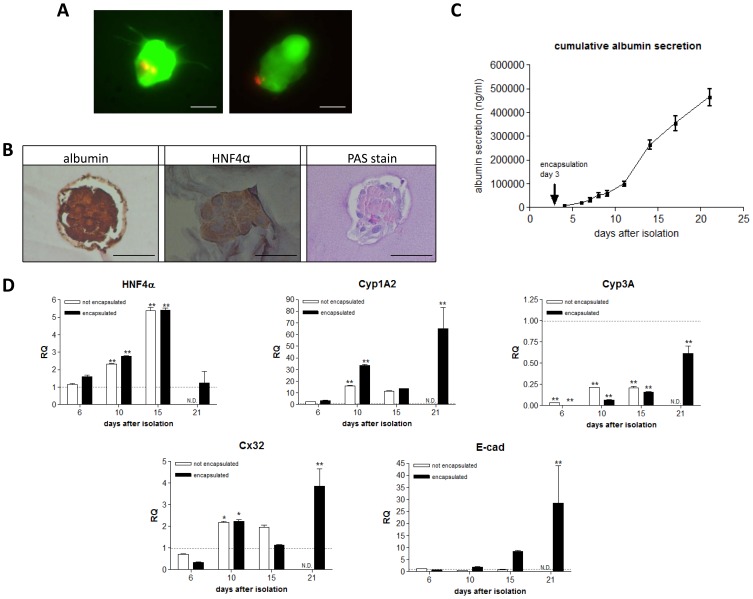
Evaluation of micro-aggregates after encapsulation in a gelatin hydrogel. (**A**) Live/dead stain of aggregates encapsulated in gelatin hydrogel at day 10. *scale bar = 50* *µm* (**B**) Stainings of the immobilized micro-aggregates at day 10 after isolation. The pictures represent stainings for albumin and HNF4α and PAS staining to visualize glycogen storage. *Pictures were recorded at 20 x magnification at day 10, scale bar = 50* *µm*. (**C**) Cummulative albumin secretion deterimend by ELISA. Albumin secretion of the immobilized cells was evaluated during 21 days of cultivation by ELISA. *Data are mean ± SD, n = 3*. (**D**) Real-time PCR for gene expression analysis after immobilization in the hydrogel. Gene expression levels of HNF4α, Cyp3A4, Cyp1A2, E-cadherin and connexin 32 (Cx32) were determined for not immobilized (white bars, not encapsulated) and immobilized micro-aggregates (black bars, encapsulated) at day 3, 6, 10 and 15 after isolation. The expression values were normalized using GAPDH as stable housekeeping gene and compared to aggregate values before encapsulation (day 3, value = 1). Data are mean RQ ± SD, n = 3. *p<0.05; **p<0.01

Therefore aggregates (φ 95±13 µm) were encapsulated and cultured in a methacrylamide-gelatin hydrogel and gene expression and cell metabolism were compared with non-encapsulated aggregates. The viability of the immobilized aggregates compared to the non-encapsulated aggregates indicated that primary hepatocytes were not affected by the encapsulation procedure. Most of the aggregates maintained their rounded morphology while some cellular outgrowth was observed at day 10 of culture (fig. 4A and B).

Similar gene expression profiles (fig 4D) between encapsulated and non-encapsulated aggregates were found, suggesting that gelatin encapsulation did not affect hepatocyte functions.

Immunohistochemistry on encapsulated aggregates demonstrate that expression of albumin and HNF4α protein was maintained during 10 days of culture. Fig. 4C depicts the cumulative albumin secretion profile of the immobilized aggregates and suggests that albumin is secreted 21 days during cell culture.

## Discussion

Currently, several cell aggregation methods have been described in literature e.g. static cell culture on non-adherent surfaces, hanging drop and rotary culture systems leading to variable aggregate dimensions mostly involving cumbersome technical handling of cells and culture media.[5], [6] The creation of large amounts of aggregates with predefined, controlled and uniform sizes in a reproducible and accurate manner is hard to control with the above-mentioned culture techniques and limits further development of aggregate based, or modular tissue engineered constructs. In this study, the potential of an agarose micro-well system has been investigated which allows controlled aggregation of primary hepatocytes into a large number of uniform cell aggregates with predefined dimensions (±50–100–180 µm) within 3 days after cell seeding and isolation from mouse livers. In addition, we confirm that HepG2 cells, a commonly used hepatocyte cell line, benefit from aggregation and three dimensional cell culture and that their function is increased in the larger aggregates used in this study. This is in line and complementary with studies reporting differentiation and increased expression of several hepatocyte specific markers (such as increased albumin secretion, urea, …) in HepG2 spheroids.[7], [17]

For the practice of aggregate based tissue engineering (e.g. the directed assembly of aggregates into larger constructs using bioprinting applications), aggregate uniformity might be important. In these approaches, the use of aggregates with predefined sizes might be desirable as well. Some papers report the improved functional performance of uniform hepatocyte aggregates, formed in PDMS or polystyrene micro-wells. However, the success of these systems was dependent on rotary culture systems and in both papers only aggregates with variable dimensions (ranging from 100–300 µm) have been used for further functional evaluation, assuming that all aggregates perform in the same way.[4], [18]

In contrast to the aforementioned studies we demonstrate that hepatocyte performance is directly affected by aggregate size and the number of cells/aggregate. While aggregates of about 200 µm diameter showed enhanced expression of hepatocyte specific markers as compared to cells cultured in monolayers, we found that hepatocyte function was further improved in 95±13 µm diameter aggregates. We observed more dead cells in the centre of the 185±16 µm diameter aggregates, as compared to the 95±13 µm aggregates. This is in contrast to others who observed no cell death in 180 µm diameter aggregates.[4] HepG2 cells were found to be less prone to cell viability effects related to the aggregate size. However, a substantial amount of dead cells was observed in aggregates with diameters exceeding 200 µm. This is in line with the knowledge that spheroids above 200 µm become hypoxic at the core. [19] For HepG2 cells, a substantial amount of dead cells was observed in the centre of the largest aggregates and this was more pronounced at day 7 as compared to day 3. Nevertheless no significant increase in aggregate diameter was observed between these time points (fig. S2). This made us suggest that the HepG2 cells did not proliferate, however we can not completely exclude this possibility since the aggregates might experience a further compactation between day 3 and day 7 while proliferating. The observation that more dead cells were present at day 7 when compared to day 3 for the larger aggregates can be explained as follows: It takes 48–72 hours to form stable aggregates, during that time the cells are more exposed to nutrients and oxygen than after compactation of the spheroid (taking place at 48–72 hours). During the next few days the oxygen and nutrient limitations become more important/limited in the larger spheroids, as a consequence more cells die within the larger aggregates at the later time point.

We postulate that differences in functional performance between 100 µm and 200 µm aggregates can be attributed to an impaired nutrient and oxygen diffusion in the largest aggregates. Our findings are in line with studies modeling mass transfer in hepatocytes indicating 100 µm diameter aggregates as ‘ideal’.[20], [21] Further decreasing the aggregate diameter to 50 µm did not improve the functional performance of hepatocytes. The dimensions of these aggregates might be too limited to create a functional three dimensional organization with sufficient cells in close contact with each other.

Gene expression of the 95±13 µm aggregate was further evaluated and compared to the gene expression of freshly isolated hepatocytes, reflecting the gene expression *in vivo*. The expression of HNF4α, an important hepatocyte specific transcription factor, remained unchanged and increased at day 10 and 15 after isolation. The expression of Cx32 and Cyp3A had decreased to 50–75%, indicating some loss of function, but significantly less than in two-dimensional cultured cells. A clear upregulation of E-cadherin, a cell adhesion molecule, expression was observed in the aggregates. This enabled cell aggregation by increasing cell-cell contacts and allowing compactation and is in line with data in literature reporting this increase as necessary for cell aggregation.[22] [23]

To use aggregates as building blocks for modular tissue engineering one needs to organize and immobilize these small aggregate structures into a larger construct while cells remain viable and maintain their appropriate function. Others have described directed assembly of cell-laden microgels for the creation of larger functional pseudo-tissues. [24], [25] Here we demonstrate that aggregates can be efficiently encapsulated in cross-linked methacrylamide-gelatin hydrogel, without interfering with cell viability and hepatocyte specific function. We found that encapsulating multiple well defined primary hepatocyte aggregates into methacrylamide gelatin hydrogel in bulk can already lead to a viable and functional construct without the need for microencapsulating each individual aggregate to form a large functional tissue. Another more practical application of predefined and controlled cell aggregation could be the use of organ specific aggregates for cell printing of large tissue constructs. Our group and others showed that by using gelatin to print hepatocytes such tissue engineered scaffolds can be made without impeding cell viability and function. Printing, or plotting, of hepatocyte aggregates as building blocks in predefined structures with high accuracy, resolution and control over pore dimensions leads to further improvement in mass transport of nutrients and metabolites.[26], [27] Our results clearly show that aggregate dimensions need to be carefully considered since aggregate size and cell number affect cell function and survival in a direct manner and that primary hepatocytes seem to function best in aggregates between 90–120 micrometer diameter.

In conclusion our findings show that the suggested agarose microwells are well suited for the large scale production of uniform hepatocyte aggregates. Moreover the results suggest that a selection of aggregate parameters might influence the outcome of the experiment. Our results also demonstrate that a modified gelatin hydrogel might be well suited for the (directed) immobilization of these aggregates in order to generate larger scale constructs for a tissue engineering approach.

## Supporting Information

Figure S1 **Schematic overview of microwell fabrication.**
(DOCX)

Figure S2 **live/dead fluorescence staining of aggregates in the 400 µm agarose chip.** Pictures, recorded at 10x magnification, represent HepG2 aggregates after 3 days (a–d) and 7 days (e–h) of cultivation in the 400 µm agarose chip at variable cell densities yielding aggregates with a diameter of 231 µm (a,e), 261 µm (b,f), 297 µm (c,g) and 307 µm (d,h).
(DOCX)

Figure S3 **live/dead fluorescence staining of aggregates in the 200 µm agarose chip.** Pictures, recorded at 20x magnification, represent HepG2 aggregates after 3 days (a–d) and 7 days (e–h) of cultivation in the 200 µm agarose chip at variable cell densities yielding aggregates with diameters of 116 µm (a,e), 142 µm (b,f), 157 µm (c,g) and 166 µm (d,h).
(DOCX)

Figure S4 **Albumin secretion of primary hepatocyte aggregates with diverse dimensions 7 days after isolation.** Albumin secretion in the cultivation medium during 24 hours is determined by ELISA and normalized using MTT assay. Data are means ± SD (n = 2).
(DOCX)

Figure S5 **Induced cytochrome 3A4 activity in aggregates of different diameter versus cells cultured on tissue culture plastic.** Cyp3A4 activity was quantified after treatment with an inducing agent (hydrocortisone) using a luciferase based assay after 7 days of isolation. *Data are mean ± SD, n = 2*.
(DOCX)

Figure S6 **Real-time PCR analysis of HepG2 cell aggregates with diverse dimensions.** Gene expression levels of albumin, TTR and HNF4α determined after 3 or 7 days of cultivation in the agarose chip. RNA levels were normalized using GAPDH as a stable housekeeping marker and the relative gene fold changes, compared to the gene expression of the control culture (TCP plated cells), were determined using the 2^−ΔΔCt^ method. Data are mean RQ ± SD, n = 3.
(DOCX)

Figure S7 **Albumin secretion of HepG2 cell aggregates with diverse dimensions.** Albumin secretion in the cultivation medium during 24 hours is determined by ELISA and normalized using MTT assay. Data are means ± SEM (n = 3).
(DOCX)

Figure S8 **Transmission electron micrographs of primary hepatocytes cultured for 10 days as aggregates.** The cytoplasm of the cells displays numerous mitochondria and an abundant RER (A). Adjoining cells show narrow contacts between the cells and the presence of junctional structures as desmosomes (B) and gap junctions (C).
(DOCX)

Table S1 **Primer sequences for isolated mouse hepatocytes.**
(DOCX)

Table S2 **Primer sequences for HepG2 cells.**
(DOCX)
