# Correction: High Throughput Micro-Well Generation of Hepatocyte Micro-Aggregates for Tissue Engineering

**DOI:** 10.1371/journal.pone.0114315

**Published:** 2014-11-19

**Authors:** The PLOS ONE Staff

There is an error in Table 1. The values in the bottom four rows of the Mean number of cells/aggregate column are incorrect. Please see the correct Table 1 here.

**Table 1:** Overview of the applied parameters for HepG2 cells and primary hepatocytes and their characteristics.

Seeded number of cells/chip	Mean aggregate diameter (µm)	Mean number of cells/aggregate	Mean aggregate volume (µm^3^)	Diameter of micro-wells in the chip
1000000	307 ± 13	629	15120449	400 µm
750 000	297 ± 9	472	13745009	400 µm
500 000	261 ± 15	314	9255967	400 µm
250 000	231 ± 7	157	6479261	400 µm
1 000 000	166 ± 11	349	2408105	200 µm
750 000	157 ± 9	262	2010823	200 µm
500 000	142 ± 7	165	1489732	200 µm
250 000	116 ± 9	87	810959	200 µm
