# Supplementary figures and images for: High Throughput Micro-Well Generation of Hepatocyte Micro-Aggregates for Tissue Engineering
Source: PLoS One. 2014 Aug 18;9(8):e105171. doi: 10.1371/journal.pone.0105171 (PMC4136852; doi:10.1371/journal.pone.0105171)

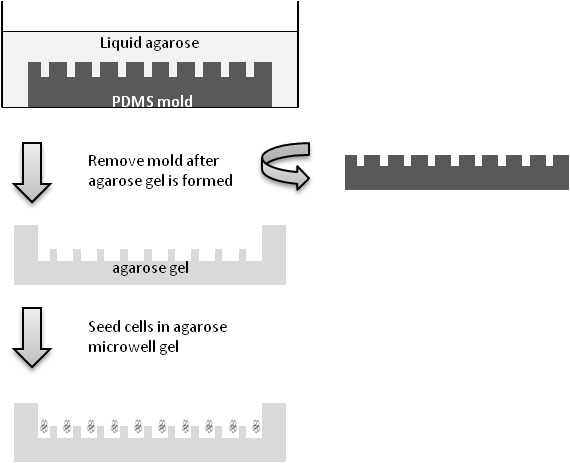


**Figure S1. Schematic overview of microwell fabrication**

Supplement: Figure S1 — Schematic overview of microwell fabrication. (DOCX) [file pone.0105171.s001.docx]
