# Supplementary material for: High Throughput Micro-Well Generation of Hepatocyte Micro-Aggregates for Tissue Engineering
Source: PLoS One. 2014 Aug 18;9(8):e105171. doi: 10.1371/journal.pone.0105171 (PMC4136852; doi:10.1371/journal.pone.0105171)
Supplement: Figure S2 — live/dead fluorescence staining of aggregates in the 400 µm agarose chip. Pictures, recorded at 10x magnification, represent HepG2 aggregates after 3 days (a–d) and 7 days (e–h) of cultivation in the 400 µm agarose chip at variable cell densities yielding aggregates with a diameter of 231 µm (a,e), 261 µm (b,f), 297 µm (c,g) and 307 µm (d,h). (DOCX) [file pone.0105171.s002.docx]

**Figure S2. live/dead fluorescence staining of aggregates in the 400 µm agarose chip.** Pictures, recorded at 10x magnification, represent HepG2 aggregates after 3 days (a-d) and 7 days (e-h) of cultivation in the 400 µm agarose chip at variable cell densities yielding aggregates with a diameter of 231 µm (a,e), 261 µm (b,f), 297 µm (c,g) and 307 µm (d,h).
