# Supplementary material for: High Throughput Micro-Well Generation of Hepatocyte Micro-Aggregates for Tissue Engineering
Source: PLoS One. 2014 Aug 18;9(8):e105171. doi: 10.1371/journal.pone.0105171 (PMC4136852; doi:10.1371/journal.pone.0105171)
Supplement: Figure S3 — live/dead fluorescence staining of aggregates in the 200 µm agarose chip. Pictures, recorded at 20x magnification, represent HepG2 aggregates after 3 days (a–d) and 7 days (e–h) of cultivation in the 200 µm agarose chip at variable cell densities yielding aggregates with diameters of 116 µm (a,e), 142 µm (b,f), 157 µm (c,g) and 166 µm (d,h). (DOCX) [file pone.0105171.s003.docx]

**Figure S3. live/dead fluorescence staining of aggregates in the 200 µm agarose chip.** Pictures, recorded at 20x magnification, represent HepG2 aggregates after 3 days (a-d) and 7 days (e-h) of cultivation in the 200 µm agarose chip at variable cell densities yielding aggregates with diameters of 116 µm (a,e), 142 µm (b,f), 157 µm (c,g) and 166 µm (d,h).
