# Supplementary material for: High Throughput Micro-Well Generation of Hepatocyte Micro-Aggregates for Tissue Engineering
Source: PLoS One. 2014 Aug 18;9(8):e105171. doi: 10.1371/journal.pone.0105171 (PMC4136852; doi:10.1371/journal.pone.0105171)
Supplement: Figure S4 — Albumin secretion of primary hepatocyte aggregates with diverse dimensions 7 days after isolation. Albumin secretion in the cultivation medium during 24 hours is determined by ELISA and normalized using MTT assay. Data are means ± SD (n = 2). (DOCX) [file pone.0105171.s004.docx]

**Figure S4. Albumin secretion of primary hepatocyte aggregates with diverse dimensions 7 days after isolation**. Albumin secretion in the cultivation medium during 24 hours is determined by ELISA and normalized using MTT assay. Data are means ± SD (n=2).
