# Supplementary material for: High Throughput Micro-Well Generation of Hepatocyte Micro-Aggregates for Tissue Engineering
Source: PLoS One. 2014 Aug 18;9(8):e105171. doi: 10.1371/journal.pone.0105171 (PMC4136852; doi:10.1371/journal.pone.0105171)
Supplement: Figure S5 — Induced cytochrome 3A4 activity in aggregates of different diameter versus cells cultured on tissue culture plastic. Cyp3A4 activity was quantified after treatment with an inducing agent (hydrocortisone) using a luciferase based assay after 7 days of isolation. Data are mean ± SD, n = 2. (DOCX) [file pone.0105171.s005.docx]

**Figure S5. Induced cytochrome 3A4 activity in aggregates of different diameter versus cells cultured on tissue culture plastic.** Cyp3A4 activity was quantified after treatment with an inducing agent (hydrocortisone) using a luciferase based assay after 7 days of isolation. *Data are mean ± SD, n=2*
