# Supplementary material for: High Throughput Micro-Well Generation of Hepatocyte Micro-Aggregates for Tissue Engineering
Source: PLoS One. 2014 Aug 18;9(8):e105171. doi: 10.1371/journal.pone.0105171 (PMC4136852; doi:10.1371/journal.pone.0105171)
Supplement: Figure S6 — Real-time PCR analysis of HepG2 cell aggregates with diverse dimensions. Gene expression levels of albumin, TTR and HNF4α determined after 3 or 7 days of cultivation in the agarose chip. RNA levels were normalized using GAPDH as a stable housekeeping marker and the relative gene fold changes, compared to the gene expression of the control culture (TCP plated cells), were determined using the 2−ΔΔCt method. Data are mean RQ ± SD, n = 3. (DOCX) [file pone.0105171.s006.docx]

**Figure S6. Real-time PCR analysis of HepG2 cell aggregates with diverse dimensions**. Gene expression levels of albumin, TTR and HNF4α determined after 3 or 7 days of cultivation in the agarose chip. RNA levels were normalized using GAPDH as a stable housekeeping marker and the relative gene fold changes, compared to the gene expression of the control culture (TCP plated cells), were determined using the 2^-ΔΔCt^ method. Data are mean RQ ± SD, n=3.
