# Supplementary material for: High Throughput Micro-Well Generation of Hepatocyte Micro-Aggregates for Tissue Engineering
Source: PLoS One. 2014 Aug 18;9(8):e105171. doi: 10.1371/journal.pone.0105171 (PMC4136852; doi:10.1371/journal.pone.0105171)
Supplement: Figure S8 — Transmission electron micrographs of primary hepatocytes cultured for 10 days as aggregates. The cytoplasm of the cells displays numerous mitochondria and an abundant RER (A). Adjoining cells show narrow contacts between the cells and the presence of junctional structures as desmosomes (B) and gap junctions (C). (DOCX) [file pone.0105171.s008.docx]

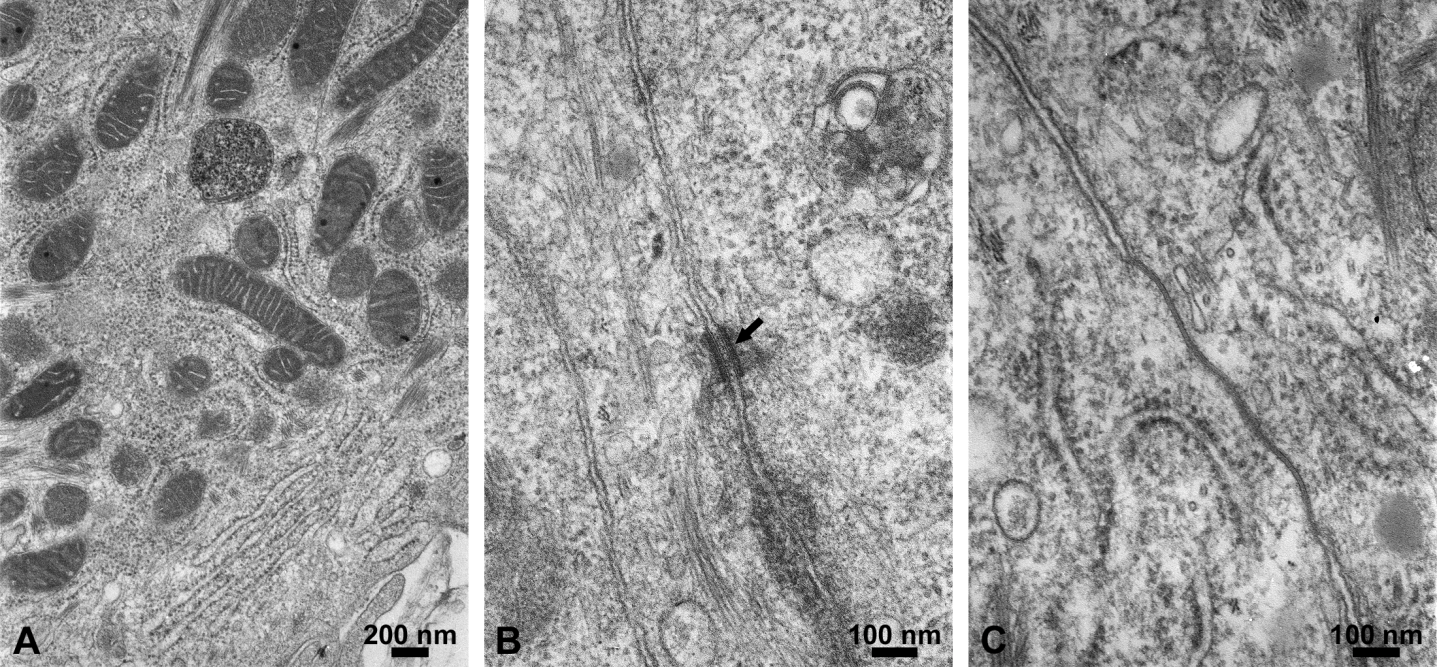


**Figure S8. Transmission electron micrographs of primary hepatocytes cultured for 10 days as aggregates.** The cytoplasm of the cells displays numerous mitochondria and an abundant RER (A). Adjoining cells show narrow contacts between the cells and the presence of junctional structures as desmosomes (B) and gap junctions (C).
