# Supplementary material for: High Throughput Micro-Well Generation of Hepatocyte Micro-Aggregates for Tissue Engineering
Source: PLoS One. 2014 Aug 18;9(8):e105171. doi: 10.1371/journal.pone.0105171 (PMC4136852; doi:10.1371/journal.pone.0105171)
Supplement: Table S1 — Primer sequences for isolated mouse hepatocytes. (DOCX) [file pone.0105171.s009.docx]

Table S1. Primer sequences for isolated mouse hepatocytes

| **Target gene** | **forward sequence (5’- 3’)** | **reverse sequence (5’- 3’)** |
| --- | --- | --- |
| *ALB* | TCTTCGTCTCCGGCTCTG | CTGGCAACTTCATGCAAAT |
| *GAPDH* | ACCCAGAAGACTGTGGATGG | ACACATTGGGGGTAGGAACA |
| *CX32* | GTGGACCTATGTCATCAGTGTGG | GGAAGGCTTCACACTTGACCAG |
| *ECAD* | AAACTTGGGGACAGCAACATCAG | TCTTTTGGTTTGCAGAGACAGGG |
| *CYP3A* | TGCTCTTCACCATGACCCACAG | CCTCATGCCAATGCAGTTCCTG |
| *HNF4α* | TGCGAACTCCTTCTGGATGACC | CAGCACGTCCTTAAACACCATGG |
| *CYP1A2* | CATCACAAGTGCCCTGTTCAAGC | AATGCTCCAGGTGATGGCTGTG |
