# Supplementary material for: High Throughput Micro-Well Generation of Hepatocyte Micro-Aggregates for Tissue Engineering
Source: PLoS One. 2014 Aug 18;9(8):e105171. doi: 10.1371/journal.pone.0105171 (PMC4136852; doi:10.1371/journal.pone.0105171)
Supplement: Table S2 — Primer sequences for HepG2 cells. (DOCX) [file pone.0105171.s010.docx]

Table S2. Primer sequences for HepG2 cells

| **Target gene** | **forward sequence (5’-3’)** | **reverse sequence (5’- 3’)** |
| --- | --- | --- |
| *GAPDH* | GAGTCAACGGATTTGGTCGT | GACAAGCTTCCCGTTCTCAG |
| *ALB* | TGGCACAATGAAGTGGGTAA | CTGAGCAAAGGCAATCAACA |
| *TTR* | ATCCAAGTGTCCTCTGATGGT | GCCAAGTGCCTTCCAGTAAGA |
| *HNF4α* | TGTACTCCTGCAGATTTAGCC | CTGTCCTCATAGCTTGACCT |
